# LISE: a server using ligand-interacting and site-enriched protein triangles for prediction of ligand-binding sites

**DOI:** 10.1093/nar/gkt300

**Published:** 2013-04-22

**Authors:** Zhong-Ru Xie, Chuan-Kun Liu, Fang-Chih Hsiao, Adam Yao, Ming-Jing Hwang

**Affiliations:** ^1^Institute of Biomedical Sciences, Academia Sinica, Taipei 115, Taiwan and ^2^National Center for Genome Medicine, Academia Sinica, Taipei 115, Taiwan

## Abstract

LISE is a web server for a novel method for predicting small molecule binding sites on proteins. It differs from a number of servers currently available for such predictions in two aspects. First, rather than relying on knowledge of similar protein structures, identification of surface cavities or estimation of binding energy, LISE computes a score by counting geometric motifs extracted from sub-structures of interaction networks connecting protein and ligand atoms. These network motifs take into account spatial and physicochemical properties of ligand-interacting protein surface atoms. Second, LISE has now been more thoroughly tested, as, in addition to the evaluation we previously reported using two commonly used small benchmark test sets and targets of two community-based experiments on ligand-binding site predictions, we now report an evaluation using a large non-redundant data set containing >2000 protein–ligand complexes. This unprecedented test, the largest ever reported to our knowledge, demonstrates LISE’s overall accuracy and robustness. Furthermore, we have identified some hard to predict protein classes and provided an estimate of the performance that can be expected from a state-of-the-art binding site prediction server, such as LISE, on a proteome scale. The server is freely available at http://lise.ibms.sinica.edu.tw.

## INTRODUCTION

Once a protein’s 3D structure becomes available, our knowledge about the protein and our ability to use the knowledge gained can be greatly enhanced. One approach for obtaining knowledge from structures is the prediction of binding sites for small molecule ligands that is needed in a variety of structure-based investigations, notably virtual docking in computer-aided drug design. Mainly because of this need, an increasing number of web servers and databases have been established for predicting/archiving small molecule-binding sites. They include those identifying the largest cavities on protein surfaces, those computing energetically favoured binding regions, and those using other computational methodologies (1–10). However, although these servers/databases have been useful in structural and functional genomics studies, their performance on a large set of protein structures remains largely unknown. This is because most of the binding site prediction methods have been tested on two small benchmark data sets containing, respectively, 210 and 48 pairs of ligand-bound and -unbound protein structures (6–10).

We recently reported a new ligand-binding site prediction method, LISE (11), in which the prediction is made based on triplets of protein surface atoms, called protein triangles, that are statistically enriched at ligand-binding sites (12). A brief description of the methodology is provided later in the text; details of the methodology and all its parameters and how they were determined can be found in our previous publications (11,12). We modelled potential ligand-binding sites as spheres of a fixed size (5.5 Å radius) containing regularly spaced grid points on protein surface. For each grid point, we extracted its surrounding protein triangles, which are triangles formed by three non-hydrogen protein surface atoms that are confined in certain triangular geometries determined from a statistical analysis of 6276 protein–ligand complex structures. These triangles were classified into various types based on chemical nature of their constituent atoms, and each type had a statistically derived propensity value to be present at ligand-binding sites. The geometric/network motifs mentioned in abstract refer to these triangles. By summing the propensity values of its surrounding triangles, each grid point received a grid score; by summing the grid scores of its grid points, each sphere received a sphere score. The spheres (i.e. predicted ligand-binding sites) were then ranked by sphere scores.

We now present the LISE server, which allows users to retrieve LISE’s pre-computed results for 63 783 protein structures or request a new prediction on structures provided in PDB (13) format. LISE’s performance using the two commonly used small benchmark data sets and targets of two critical assessment of protein structure prediction experiments (14,15) has been previously reported (11). The evaluation has now been extended to a large set of non-redundant protein–ligand complex structures compiled by Brylinski and Skolnick (3). To our knowledge, this is the largest test (2073 structures evaluated) ever reported evaluating ligand-binding site predictions.

## MATERIALS AND METHODS

### Data sets of protein structures

We downloaded the biological units of protein structures from the ftp site of the PDB (13) on 14 March 2012. The number of entries (i.e. PDB format files) downloaded was 72 106. For those with multiple biological unit files (i.e. pdb1, .pdb2 and so forth), the first file was used. Those containing nucleic acid fragments or not containing coordinates of protein atoms were ignored, leaving 63 783 structures for LISE to predict their ligand-binding sites regardless of whether the structure was a complex containing a bound ligand molecule. The results were stored as pre-computed predictions, which can be retrieved when an input PDB ID matches that of any of the 63 783 structures computed.

To further extend the evaluation of LISE beyond that described in our previous study (11), we also downloaded a non-redundant data set of protein–ligand complex structures from the FINDSITE website (3). In this data set, a PDB entry may be separated into several different files, each containing only a single protein chain and, as a result, the predictions can contain many duplications. Furthermore, as biological units should be used to avoid missing the opportunity of predicting binding sites located at the interface between multiple protein chains (11), the same PDB IDs of these FINDSITE entries were used to download their biological units from the PDB website to evaluate LISE’s performance. The exclusion of ∼60 entries in which the ligands were small solvent molecules, such as 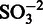 or 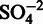, left 2073 ligand-bound protein structures to be used for evaluating LISE.

### Input and output

**Figure 1.**
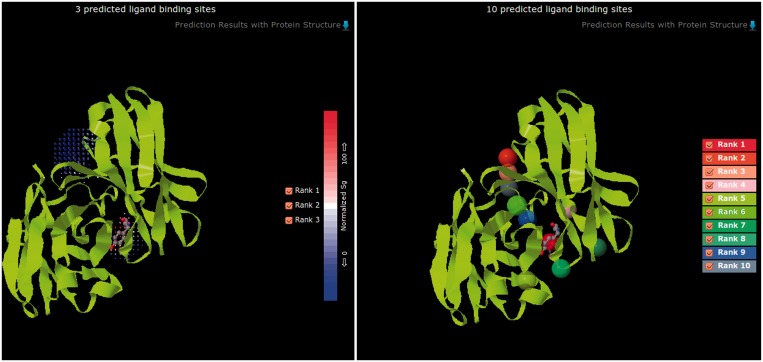
Display of LISE-predicted ligand-binding sites. LISE’s prediction results are displayed as (left) the Top3 predicted sites with their grid points colour-coded according to binding site-enrichment score (11) and (right) the Top10 predicted sites represented by spheres colour-coded according to their predicted rank. Users can also view individual site(s) separately by ticking their rank box.

The LISE server was written in Java language. A Java script was used to take the input, extract protein sequences and atom coordinates and execute PSI-BLAST (16) (to generate residue conservation scores to be used by LISE) and other routines of the LISE method (11). The resulting files were parsed and combined, and the predictions were displayed on two windows of the interactive browser plugin, Jmol (17). One window displayed the Top3 predicted sites, each represented by a cluster of tiny grid points, and the other the Top10 predicted sites, each represented by a sphere (Figure 1). There is an option to download the coordinates of these grid points and sphere centres in a PDB format output file in which these binding sites coordinates are added to the original structure coordinates file.

All computations and file handlings have been automated and users need only provide an input, which can be either a PDB ID or the uploading of a protein structure in PDB format. When the input is a PDB ID matching one of the 63 783 pre-computed structures, prediction results will be immediately displayed (see Figure 1 for an example). When the input is an upload of a PDB structure file, the server will carry out a new LISE prediction, which may take several minutes or longer to complete, and a web link will be provided for users to check the progress of the computation. The server does not require a login.

On a PC with four 2 GHz CPUs, the LISE algorithm took ∼7 min to complete the computation for PDB 1a6w (229 residues) and ∼47 min for PDB 3dao (539 residues). The algorithm has a time complexity of n^2^ (n refers to the number of protein surface atoms) because it computes distances between any two surface atoms of the target protein. When the size of the protein or its biological unit becomes very large, such as that of a virus capsid, it could take a week for LISE to finish the prediction, and longer if there are other jobs running on the server at the same time. Users are, therefore, advised to input PDB ID for pre-computed results or structure files of a single protein chain for large proteins. A stand-alone version of LISE is also available for download from the server.

## RESULTS AND DISCUSSION

### Evaluation of the prediction results

LISE has been shown to achieve significantly better Top1 (>80%) and Top3 (>90%) success rates than many other methods when tested on two commonly used small benchmark data sets (11). For the much larger non-redundant set of 2073 protein–ligand complexes retrieved from the FINDSITE website (3) (see ‘Materials and Methods’ section), LISE’s Top1 and Top3 success rates were 72.7 and 85.6%, respectively (Table 1), both showing a decrease of ∼10% compared with those using the small data sets. However, they are still significantly better than the reported Top5 success rates for FINDSITE (70.9%) and LIGSITE^csc^ (∼50%) on a subset (901 complexes) of the large non-redundant data set (3).

**Table 1.** LISE success rates for different functional categories of proteins^a^

Protein category^b^	Number of proteins	Top1 success rate (%)	Top3 success rate (%)
Hormone-binding protein	23	17	48
Kinase	112	49	67
Membrane protein	22	64	73
Transcription-related protein	38	37	75
Unknown function	46	65	78
Specific molecule (e.g. sugar/lipid/odorant) binding protein	99	66	81
Transport protein	110	73	82
Chaperone	22	75	82
Transferase	311	69	85
Oxidoreductase	230	77	90
Immune system	30	60	90
Hydrolase	615	84	91
Signalling protein	32	72	91
Lyase	85	80	94
Isomerase	66	88	95
Ligase	41	83	95
All other categories (<20 structures in each category)	191	62	78
Overall	2073	72.7	85.6

^a^Success rates were computed as the percentage of the query structures in a category for which the best (Top1) or any one of the best three (Top3) predicted binding sites satisfied the distance criterion (shortest distance between the centre of the predicted site and the bound ligand’s non-hydrogen atoms <4 Å).

^b^Classified according to the molecular description included in the HEADER section of each PDB file.

To examine possible factors contributing to the lower success rates of LISE using the large data set, proteins were grouped into functional categories based on the molecular description included in the HEADER section of each PDB entry. As shown in Table 1, LISE’s success rates for several functional categories, namely, hormone-binding proteins, kinases and transcription-related proteins, were significantly worse than for others, especially in the case of the Top1 success rates. Two possible explanations are described later in the text.

### Kinases

**Figure 2.**
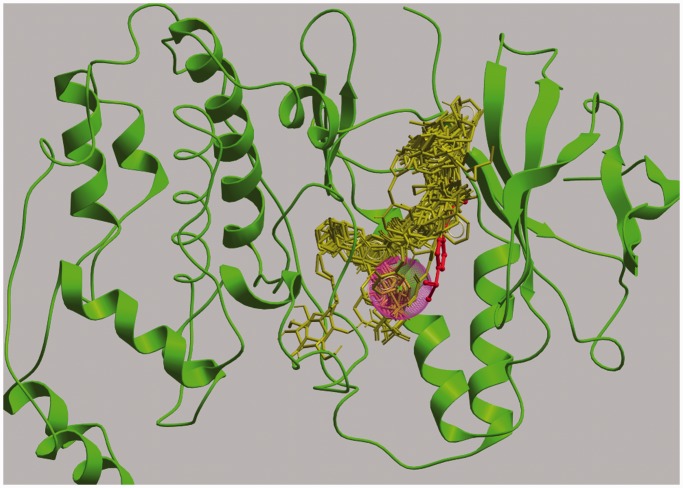
The binding of ligands in the binding site pocket of various kinases. The Figure shows a superimposition of 81 kinase–ligand complex structures downloaded from the Pocketome server (19), but, for clarity, only one protein structure, MAP kinase p38 (PDB ID: 1a9u), is shown (green ribbons). Ligand molecules are shown as sticks; that for MAP kinase p38 is shown in red and all others in yellow. LISE’s Top1 predicted site (purple sphere) for 1a9u is close to its ligand (red stick), but not close enough to be determined as a successful prediction by the <4 Å distance criterion. This figure was created using the ICM browser (20).

Except for kinases, it is relatively easier for LISE to predict ligand-binding sites of enzymes than those of other types of proteins, as clearly shown in Table 1. Most kinases belong to a family of proteins that, although having similar 3D structures, bind a variety of ligand molecules in overlapping regions, which, when taken together, form a long, narrow binding site pocket (18) (Figure 2). As we have discussed previously (11), the binding site predicted by LISE, which is represented by a rather small sphere, does not cover the entire pocket and, as a result, often fails to ‘hit’ a specific ligand molecule within the distance required to be determined as a successful prediction (Figure 2). Nevertheless, at least in the case of kinases, the low success rate of LISE’s prediction should not diminish its usefulness in most applications, such as docking computations, as the predicted binding site sphere is usually located within the large binding site pocket. For example, visual inspection of the 112 kinase structures computed (Table 1) showed that for 101 (90%) of them, LISE’s Top1 sphere was inside the binding site pocket. However, there is room for improvement in LISE’s prediction specificity for protein families with a large binding site pocket, such as kinases. A more informative and useful way to compute binding site prediction success rates taking into account multiple protein structures and diverse ligands is also desirable.

### Data set composition

The binding site scoring of LISE was developed from a statistical analysis of >6000 protein–ligand complex structures from the PDB (11,12). About 70% of these structures are enzymes, reflecting a bias in the experimental determination of protein–ligand complex structures for the purpose of, for example, drug development. Such a bias towards enzymes is also evident in the non-redundant test data set in which transferases, oxidoreductases and hydrolases were even more abundant than kinases (Table 1). In fact, 80% of the 210 structures commonly used as a benchmark data set for ligand-binding site predictions (8) are enzymes, and some of these structures are similar in proteins belonging to the same family (e.g. kinases), even though their amino acid sequences have diverged. With such a bias and as a result of the situation with kinases described earlier in the text, it is not surprising that LISE performed particularly well for enzymes. It remains to be determined whether other prediction methods suffer the same data set bias, but our evaluation points out the need for caution in interpreting the success rates of ligand-binding site predictions.

## SUMMARY

In this article, we introduced the LISE web server for a novel ligand-binding site prediction method for any given protein 3D structure. The server takes a PDB ID or a PDB format structure file and, by an automated process, displays/outputs Top3 and Top10 predicted sites. Tests on commonly used benchmark data sets and on a very large non-redundant data set indicated that the new server provides accurate and reliable predictions of protein ligand-binding sites. Therefore, the new server can help in virtual drug design and other structure-based studies, although considerably lower success rates can be expected for some particular types of proteins, such as hormone carriers or hormone-binding proteins.

## FUNDING

National Science Council of Taiwan [NSC 96-2627-B-001-004 and NSC 100-2811-B-001-067 to Z.R.X.]. Funding for open access charge: Institute of Biomedical Sciences Academia of Sinica, Taiwan.

*Conflict of interest statement*. None declared.
